# Cardiac Echinococcosis, an Unusual Echocardiographic Finding

**DOI:** 10.4269/ajtmh.2010.09-0557

**Published:** 2010-02-05

**Authors:** Juan Cataño

**Affiliations:** Facultad de Medicina, Universidad de Antioquia, Medellín, Colombia

**Figure 1.**
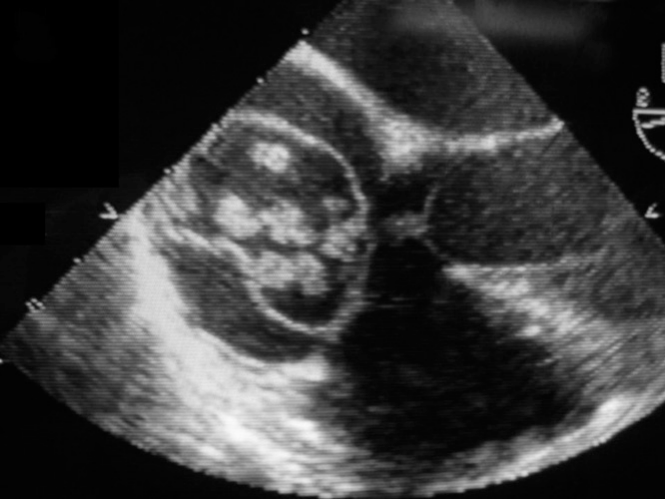
Initial manifestations of the patient with transesophageal echocardiography, showing a 4 × 3 cm cyst with multiple internal structures.

**Figure 2.**
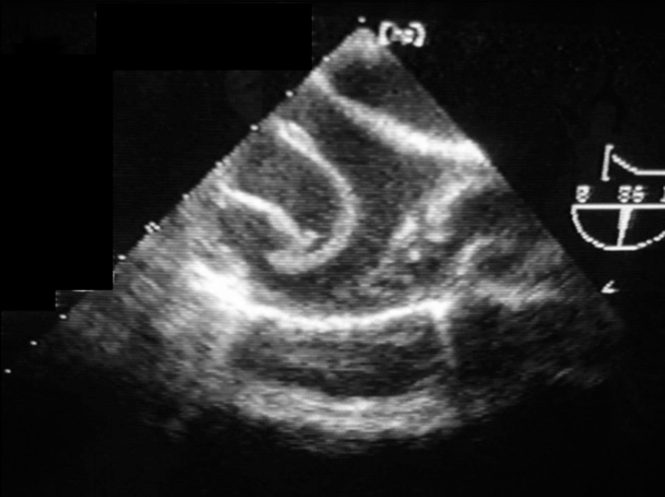
Second transesophageal echocardiography performed on the patient after abrupt onset of chest pain and fever, showing the cyst wall without the multiple, internal, small, kidney-shaped cystic lesions consistent with spontaneous cyst rupture.

Cardiac echinococcosis is an unusual echocardiographic finding.^1^ An 18-year-old woman from Colombia, who had lived her entire life on a farm and had no remarkable medical history, was admitted to a hospital because of three weeks of non-specific upper abdominal pain and intermittent jaundice. There was no fever, chest pain, palpitation, dysnea, or other cardiac symptoms. Results of a heart examination were normal. Her laboratory data, including blood chemistries, electrocardiogram, and chest radiograph, were normal. Transesophageal echocardiography^2^ was performed because chest computed tomography showed an atrial mass, a 4 × 3 cm cyst with multiple and mobile small internal structures, in the right atrium. This finding was consistent with a cardiac hydatid cyst (Figure 1). Serologic analysis confirmed the diagnosis. Treatment with albendazole, 200 mg every 12 hours, was started, and 1 day before scheduled surgery she had fever. Cardiac complications were suspected because of the presence of the cyst. Therefore, another transesophageal echocardiogram was performed and showed a right atrial cyst without multiple internal mobile small kidney-shaped cystic lesions, which suggested cyst rupture with multiple cyst embolisms (Figure 2). Surgery was canceled and the patient responded well to albendazole treatment alone without recurrence of symptoms.
